# Thermodynamic Modeling of Poorly Complexing Metals in Concentrated Electrolyte Solutions: An X-Ray Absorption and UV-Vis Spectroscopic Study of Ni(II) in the NiCl_2_-MgCl_2_-H_2_O System

**DOI:** 10.1371/journal.pone.0119805

**Published:** 2015-04-17

**Authors:** Ning Zhang, Joël Brugger, Barbara Etschmann, Yung Ngothai, Dewen Zeng

**Affiliations:** 1 College of Chemistry and Chemical Engineering, Central South University, Changsha 410083, P. R. China; 2 School of Chemical Engineering, The University of Adelaide, Adelaide 5000, South Australia, Australia; 3 Division of Mineralogy, South Australian Museum, Adelaide 5000, South Australia, Australia; 4 School of Geosciences, Monash University, Clayton 3800, Victoria, Australia; Reader in Inorganic Chemistry, UNITED KINGDOM

## Abstract

Knowledge of the structure and speciation of aqueous Ni(II)-chloride complexes is important for understanding Ni behavior in hydrometallurgical extraction. The effect of concentration on the first-shell structure of Ni(II) in aqueous NiCl_2_ and NiCl_2_-MgCl_2_ solutions was investigated by Ni K edge X-ray absorption (XAS) and UV-Vis spectroscopy at ambient conditions. Both techniques show that no large structural change (e.g., transition from octahedral to tetrahedral-like configuration) occurs. Both methods confirm that the Ni(II) aqua ion (with six coordinated water molecules at *R*_Ni-O_ = 2.07(2) Å) is the dominant species over the whole NiCl_2_ concentration range. However, XANES, EXAFS and UV-Vis data show subtle changes at high salinity (> 2 mol∙kg^-1^ NiCl_2_), which are consistent with the formation of small amounts of the NiCl^+^ complex (up to 0.44(23) Cl at a Ni-Cl distance of 2.35(2) Å in 5.05 mol∙kg^-1^ NiCl_2_) in the pure NiCl_2_ solutions. At high Cl:Ni ratio in the NiCl_2_-MgCl_2_-H_2_O solutions, small amounts of [NiCl_2_]^0^ are also present. We developed a speciation-based mixed-solvent electrolyte (MSE) model to describe activity-composition relationships in NiCl_2_-MgCl_2_-H_2_O solutions, and at the same time predict Ni(II) speciation that is consistent with our XAS and UV-Vis data and with existing literature data up to the solubility limit, resolving a long-standing uncertainty about the role of chloride complexing in this system.

## Introduction

One of the most challenging tasks in the extractive metallurgy of nickel from ores or Ni-containing industrial wastes is its separation from Co and Cu [1,2]. Pospiech and Walkowiak [3] developed a separation method by taking advantage of the different tendency between Ni and Co to form complexes with the chloride ion [4,5]. Further development of these ionic exchange methods to remove trace Cu/Co from Ni chloride aqueous solutions require a profound understanding of the formation of Ni(II)-chloride complexes in NiCl_2_ aqueous solution.

A number of studies investigated Ni(II) aqueous solutions in the presence of excess Cl^-^ions (Cl/Ni >> 2) under a wide range of conditions using different techniques; a summary is given in Table 1. Most of these investigations [5–12] agree that the octahedral NiCl^+^ and [NiCl_2_]^0^ species are formed at ambient temperature. Other divalent transition metals such Cu(II) [13–15], Co(II) [4] and Zn(II) [16] form anionic tetrahedral-like MCl_4_^2—^complexes in the presence of a large excess of HCl or LiCl at room-temperature. In contrast, the anionic [NiCl_3_(H_2_O)]^-^complex with tetrahedral-like configuration appears to be the highest order Ni(II) chloro-complex in aqueous solutions, and was observed only at elevated temperature [5].

**Table 1:** Previous studies on aqueous Ni(II) halide complexes in aqueous solutions.

Method	T, P and salinity range*	Cl^‒^/Ni^2+^ ratio (Y) range	Species identified^a^	Ref.
X-ray absorption spectroscopy	25–434°C, 400–600 bar, 0 m < Cl_tot_ < 7.68 m, Ni_tot_ = 0.2 m	0.28 ≤ Y ≤42	O_h_-[Ni(H_2_O)_6_]^2+^, O_h_-Ni(H_2_O)_5_Cl^+^, O_h_-[NiCl_2_(H_2_O)_4_]^0^, T_d_-[NiCl_2_(H_2_O)_2_]^0^, T_d_-[NiCl_3_(H_2_O)]^-^	[5]
UV-Vis spectrophotometry	26–250°C, 0.1 kbar, Cl_tot_ ≤ 3 m, 0.05 ≤ Ni_tot_ ≤ 0.1 m	20 ≤ Y ≤ 54	Ni^2+^, NiCl^+^, [NiCl_2_]^0^, [NiCl_3_]^-^	[10]
EXAFS	25°C, P_sat_, 2 M ≤ Cl_tot_ ≤ 10 M, Ni_tot_ = 2 M	2 ≤ Y ≤ 5	O_h_-[Ni(H_2_O)_6_]^2+^, O_h_-[NiCl(H_2_O)_5_]^+^	[9]
X-ray diffraction	25°C, P_sat_, 4 M ≤ Cl_tot_ ≤ 6 M, Ni_tot_ = 2 M	2 ≤ Y ≤ 5	O_h_-[Ni(H_2_O)_6_]^2+^, O_h_-[NiCl(H_2_O)_5_]^+^, O_h_-[NiCl_2_(H_2_O)_4_]^0^	[11]
UV-Vis spectrophotometry	25–320°C, P_sat_, 0 m ≤ Cl_tot_ ≤ 11 m	Y > 10	O_h_-[Ni(H_2_O)_6_]^2+^, O_h_-[NiCl(H_2_O)_5_]^+^, O_h_-[NiCl_2_(H_2_O)_4_]^0^, O_h_-[NiCl_3_(H_2_O)_3_]^-^, O_h_-[NiCl_4_(H_2_O)_2_]^2–^, O_h_-NiCl_5_(H_2_O)^3–^, O_h_-[NiCl_6_]^4–^, T_d_-[NiCl(H_2_O)_3_]^+^, T_d_-[NiCl_2_(H_2_O)_2_]^0^, T_d_-[NiCl_3_(H_2_O)]^-^, T_d_-[NiCl_4_]^2–^	[6]
UV-Vis spectrophotometry	25°C, P_sat_, 0.12 M ≤ Cl_tot_ ≤ 13.7 M, Ni_tot_ = 0.06 M	1 ≤ Y ≤ 228	[Ni(H_2_O)_6_]^2+^, [NiCl(H_2_O)_5_]^+^, [NiCl_2_(H_2_O)_4_]^0^	[12]
Polarography and UV-Vis spectrophotometry	25°C, P_sat_, 1 M ≤ Cl_tot_ ≤ 13.9 M, Ni_tot_ = 0.03 M	33 ≤ Y ≤ 463	[Ni(H_2_O)_5_Cl]^+^	[8]
UV-Vis spectrophotometry	25°C, P_sat_, 0.12 M≤ Cl_tot_ ≤ 12.5 M, Ni_tot_ = 0.06 M	1 ≤ Y ≤ 208	[NiCl]^+^, [NiCl_2_]^0^	[7]

* m is molality and M is molarity.

^a^ Complex geometries: O_h_: octahedral; T_d_: tetrahedral.

The role and extent of chloride complexing in stoichiometric concentrated Ni(II) chloride solutions (Cl/Ni = 2) remains controversial [17–26]. There is broad agreement that the 6-fold-water-coordinated Ni(II) ion is the dominant complex in NiCl_2_ solutions up to high concentrations (4 mol∙L^-1^), a result supported by extended X-ray absorption fine structure (EXAFS) measurements [19,20,23], neutron diffraction measurements [22,24], and first principle molecular dynamics simulations [26]. However, some studies emphasize the existence of small but significant amounts of inner-sphere complexes in concentrated NiCl_2_ solutions. For example, on the basis of X-ray diffraction (XRD) studies, Magini [21] suggested the existence of about 8% NiCl^+^ complex in a 3.12 mol∙kg^-1^ NiCl_2_ solution, and Waizumi et al. [25] reported 0.4 Cl in the first coordination shell of Ni(II) at the solubility limit (5.06 mol∙kg^-1^ NiCl_2_). A recent Ni L_2,3_-edge X-ray absorption near edge structure (XANES) study showed less than ~10% Cl in the first coordination shell in a 1.5 mol∙L^-1^ NiCl_2_ solution [17]. However, the data show evidence for a distortion of the complex from octahedral symmetry, which was interpreted to reflect the presence of solvent-shared ion pairs.

Some XRD studies were also carried out on NiBr_2_ solutions to help resolve the ambiguities regarding the structure of NiCl_2_ solutions [27,28], as the Ni-Br distance is longer than the Ni-Cl distance, and Br^-^is a stronger X-ray scatter than Cl^-^. The results indicated the formation of strong inner-sphere complexes, with 0.29(3) Br^-^coordinated to Ni(II) in a 2 mol∙L^-1^ solution; this suggests that chloro-complexes exist in concentrated solutions of NiCl_2_ [27].

In this study we used a combination of X-ray absorption spectroscopy (XAS) and UV-Vis spectrophotometry to provide a molecular-level understanding of the effect of concentration on the speciation and structure of Ni(II)-chloride complexes in NiCl_2_ and NiCl_2_-MgCl_2_ solutions at concentrations ranging from dilute to the solubility limit at ambient temperature. A speciation-based mixed-solvent electrolyte (MSE) thermodynamic model is used to provide a speciation model that is consistent with most existing experimental and theoretical data on NiCl_2_-MgCl_2_ solutions. The results of our study can be used to predict Ni(II) behavior in high concentration brines for hydrometallurgical processing.

## Experimental

### Sample Preparation

NiCl_2_∙6H_2_O_(s)_, NiSO_4_∙6H_2_O_(s)_ and MgCl_2_∙6H_2_O_(s)_ (99.8% purity) from Sinoreagent, China, were used as XAS standards and for solution preparation. All Ni(II) solutions were prepared gravimetrically and made freshly before the experiments. The sample solutions were prepared by dissolving NiCl_2_∙6H_2_O_(s)_ in doubly deionized water. For the UV-Vis spectroscopic measurement, two series of solutions were prepared by gravimetrically mixing (i) deionized water with a 4.65 mol∙kg^-1^ NiCl_2_ stock solution (NiCl_2_-H_2_O system); and (ii) a 5.72 mol∙kg^-1^ MgCl_2_ + 0.05 mol∙kg^-1^ NiCl_2_ stock solution with a 0.05 mol∙kg^-1^ NiCl_2_ stock solution (NiCl_2_-MgCl_2_-H_2_O system). The chloride concentrations in the stock solutions were checked using the Ag chloride gravimetric method [76]. To suppress the hydrolysis of Ni(II), ~0.001 mol∙kg^-1^ hydrochloric acid (HCl_(l)_, Sinoreagent, China, AR) was added to all the Ni-Cl and Ni-Mg-Cl solutions; spectrographic measurements of acidified and non-acidified solutions were identical. The compositions of both systems were listed in the S1 and S2 Tables. In order to establish a reference free of Ni(II)-chloride complexes at different ionic strengths, two Ni(II) perchlorate solutions (0.05 and 4.46 mol∙kg^-1^) were prepared by mixing hexahydrate Ni(ClO_4_)_2(s)_ (Sinoreagent, China, AR) with deionized water.

### XAS measurement and analysis

The coordination environment of Ni(II) in solutions containing 1, 2, 4 and 5.05 mol∙kg^-1^ NiCl_2_ was characterized using XAS data obtained at the XAS beamline of the 1.5–2.2 GeV Beijing Synchrotron Radiation Facility (BSRF). This beamline has a Si(111) double-crystal monochromator with an energy resolution of 1.25 eV at the Ni K-edge (8333.0 eV). Data on solutions and solid samples were acquired in transmission mode. The solutions were placed in a cell that consisted of a square-shaped Kapton spacer having a thickness from 125 to 605 μm; the higher concentration of solution, the shorter the path length that was used. The liquid samples were injected into the cell with a micro-syringe. For the solid samples, a layer of powder was placed on a flat Kapton substrate.

EXAFS data were analyzed using the HORAE package [29], with amplitude and phase shifts calculated using FEFF6 [30]. The fits were performed in R-space using *k* ^3^-weighting and a Hanning window. The fit parameters include the scale factor ($S_{0}^{2}$), the energy shift (△*E*_0_), the number of oxygen (water molecule, *N*_O_) and chlorine (*N*_Cl_) in the first coordination shell of Ni(II), the bond distances *R*_O_ and *R*_Cl_, and the Debye-Waller factors for the O and Cl atoms ($\sigma_{\text{O}}^{\text{2}}$,$\sigma_{\text{Cl}}^{\text{2}}$). A value $S_{0}^{2}$ = 0.83(7) was obtained from fitting the EXAFS spectra of the two solid standards; this value is in excellent agreement with previous studies (5,31), and was used for fitting all the samples.

### UV-Vis measurements

Spectrophotometric measurements were carried out using a Shimadzu (Japan) UV-2550 double-beam spectrophotometer at room temperature. Sample solutions of the NiCl_2_-H_2_O and NiCl_2_-MgCl_2_-H_2_O systems were placed in 1 and 10 mm rectangular quartz cells, respectively. Dual-beam mode was used with the reference cell containing deionized water. The data were baseline-corrected using measurements of two water-filled cuvettes. Spectra were recorded at a 1.0 nm interval over the range 300–850 nm, and the absorbance range was typically between 0 and 3 absorbance units. The experimental error was estimated by multiple scans (5 times) of two solutions with 0.1 and 4 mol∙kg^-1^ NiCl_2_. The difference between these repeat spectra was generally less than 0.002–0.005 absorbance units.

The raw UV-Vis and XAS data are provided in the supporting information (S1–S10 Tables).

## Results and Discussion

### XAS investigation

#### XANES analysis

**Fig 1:**
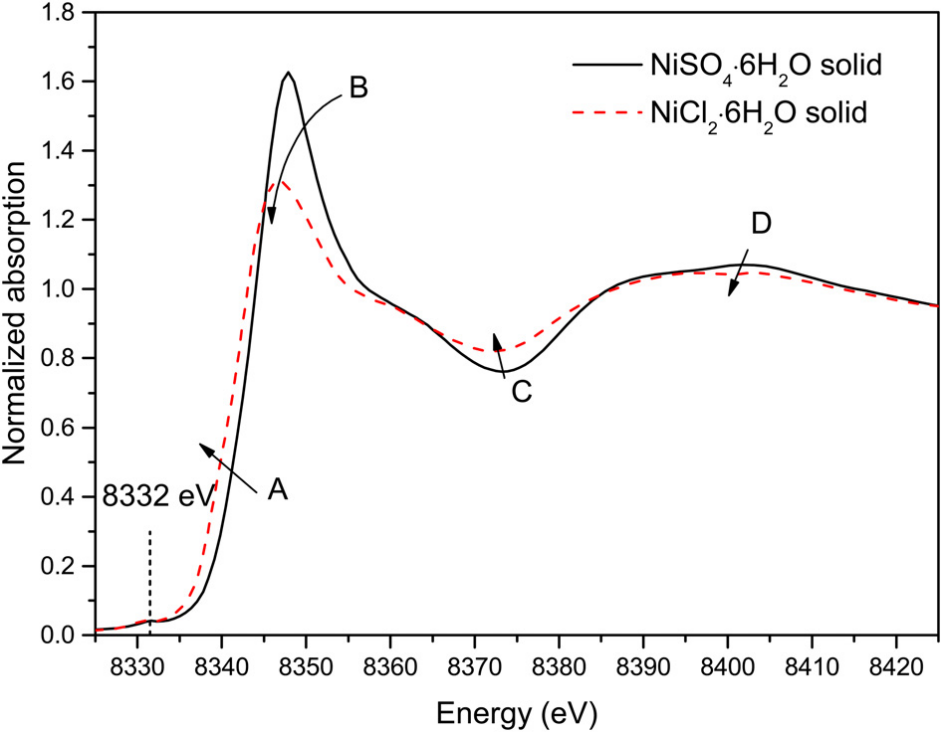
Normalized Ni K-edge XANES spectra for the two solid compounds.

Fig 1 shows the XANES spectra (Raw spectral data was collected in S1–S4 Tables) for the NiSO_4_∙6H_2_O_(s)_ and NiCl_2_∙6H_2_O_(s)_ reference compounds, which contain octahedral [Ni(H_2_O)_6_] [32] and [NiCl_2_(H_2_O)_4_] moieties [33], respectively. The main spectral differences between the two spectra are: (i) presence of a shoulder at ~8340 eV attributed to the 1*s* to 4*p* transition [31] in NiCl_2_∙6H_2_O_(s)_ (feature A); (ii) a lowering of the intensity of the white line (feature B) and shift of its position by ~ -1.2 eV in NiCl_2_∙6H_2_O_(s)_ relative to NiSO_4_∙6H_2_O_(s)_; as well as (iii) reduced intensity of the oscillation in the 8360 to 8420 eV region in NiCl_2_∙6H_2_O_(s)_ (features C and D in Fig 1).

**Fig 2:**
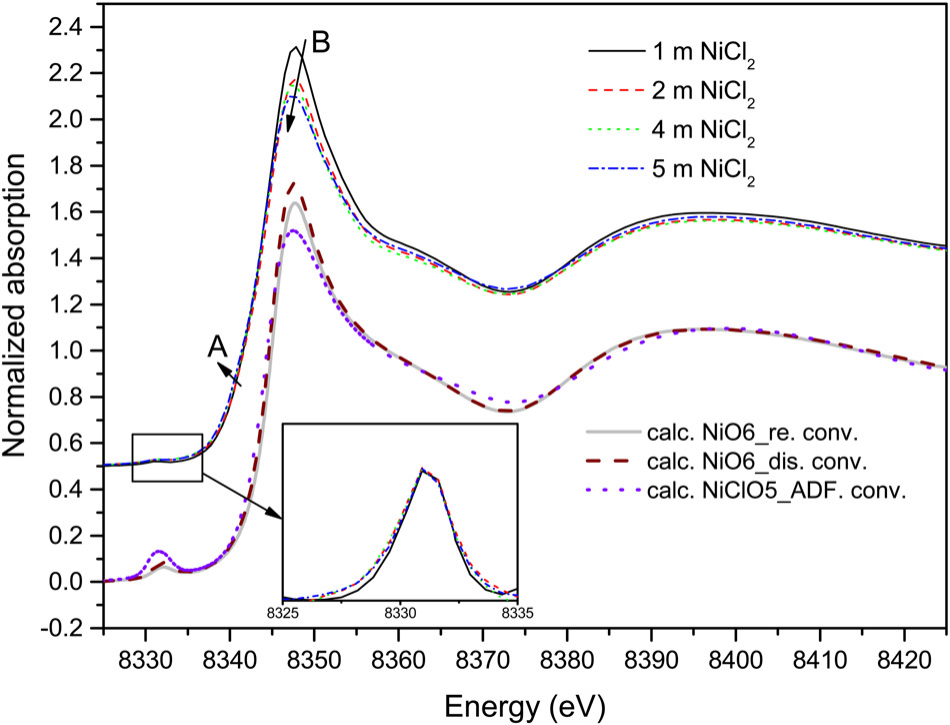
Normalized Ni K-edge XANES spectra of nickel complexes as a function of concentration (thin lines). Baseline-removed pre-edges are shown as a function of concentration in inset for clarity. Thick lines are the calculated (convoluted) XANES spectra for Ni aqueous species using fully hydrated regular (re.) and distorted (dis., DFT optimized), as well as [NiCl(H_2_O)_5_]^2+^ (ADF., DFT optimized) octahedral configuration models from literature [5].

The Ni K-edge XANES spectra (Raw spectral data was collected in S5–S8 Tables) of solutions with various NiCl_2_ concentrations at room temperature are plotted in Fig 2 with the XANES spectra calculated by Tian et al. [5] using *ab initio* XANES simulations for the NiO_6_ and NiO_5_Cl clusters. The spectra of the four solutions are similar and show features that are characteristic for octahedral transition metal complexes [5,31,34]. A slight decrease (9.7%) of the white line intensity and a subtle energy shift (0.4 eV) of the white line to lower energy (feature B in Fig 2) are observed as the NiCl_2_ concentration increases from 1 to 5.05 mol∙kg^-1^. Therefore, the changes affecting the white line region of the solution spectra are analogous with the difference of XANES spectra of the two solid reference compounds and are consistent with some Cl entering the first coordination sphere of Ni(II) upon increasing salt concentration. Similar spectral changes are confirmed by the XANES simulations of Ni clusters (Fig 2). In addition, the XANES calculations show that the differences in features C and D observed for the solid standards are due to second-shell contributions. The only significant difference between the experimental and simulated spectra lies in the pre-edge peak intensity (feature around 8332 eV), which is associated with a 1*s*→3*d* photoelectron transition and is sensitive to the local geometry of the Ni site [5,35,36]. There is no change observed in the experimental spectra (inset in Fig 2), whereas the XANES simulations predict an increase in intensity of this pre-edge upon replacement of a water molecule by a chloride ion in the Ni(II) coordination sphere. Tian et al. [5] similarly failed to confirm this feature experimentally. Overall, the XANES data are consistent with octahedral Ni(II) aqua complexes being present over the whole NiCl_2_ concentration range, with minor amounts of chloride complexing taking place at high salt concentration.

#### EXAFS analysis

**Fig 3:**
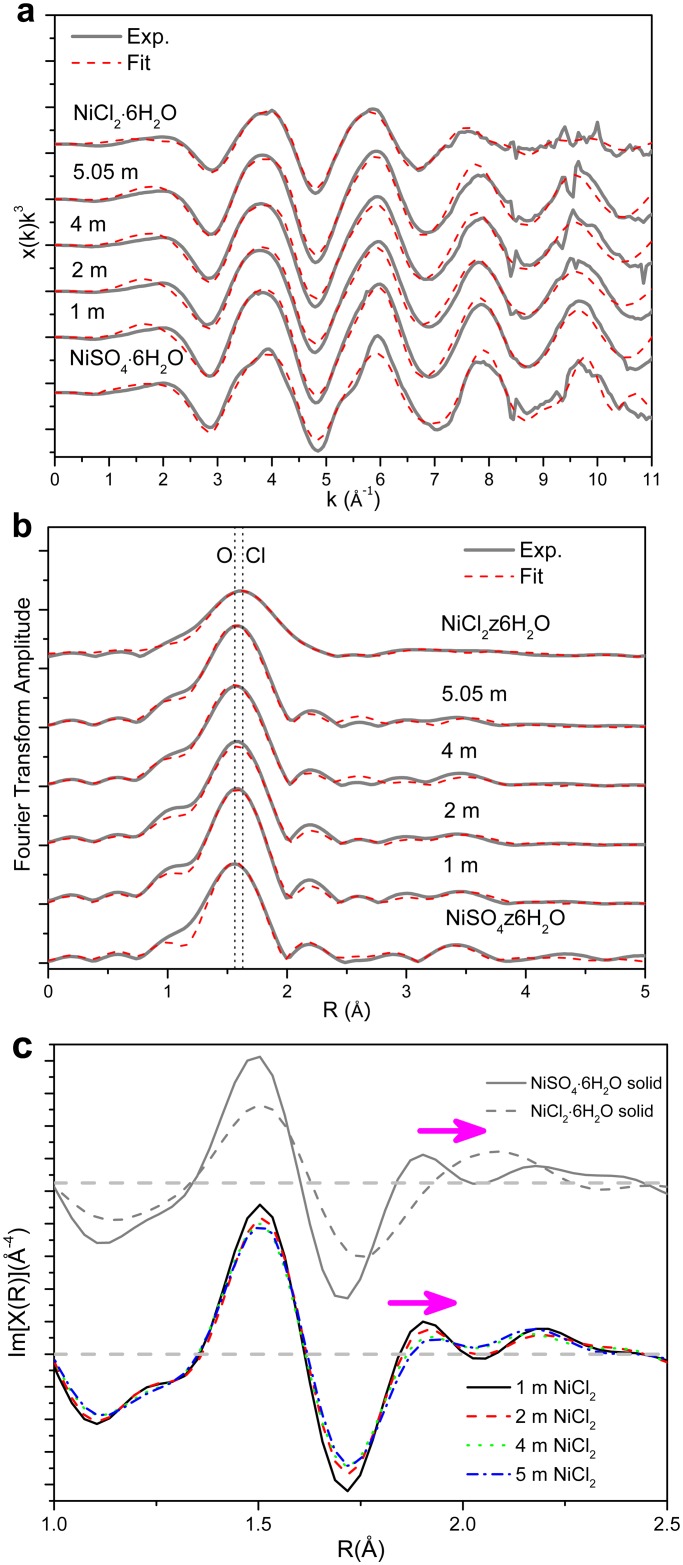
Ni K-edge *k* ^3^-weighted EXAFS (a), and the amplitude (b) and imaginary part (c) of their Fourier transforms of the two solid compounds and four NiCl_2_ solutions. The dot line is the position of Ni-O and Ni-Cl peaks.

**Fig 4:**
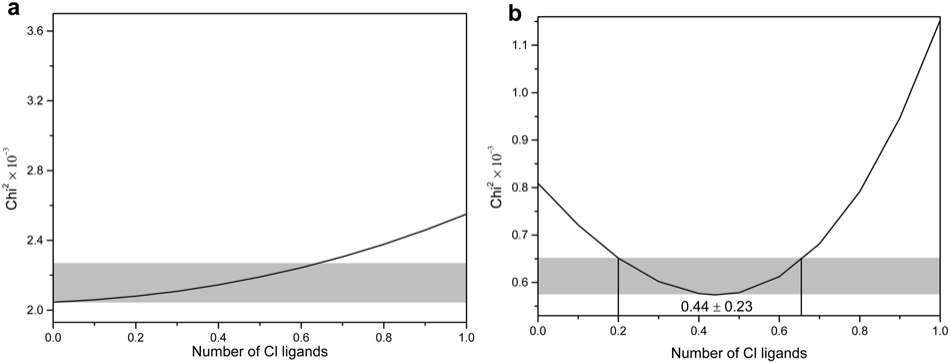
The distribution maps of analysis error of XAS spectra for 2 mol∙kg^-1^ (a) and 5.05 mol∙kg^-1^ (b) NiCl_2_ solution as a function of number of Cl ligands. The gray area represents 90% confidence level.

The Ni K-edge EXAFS *k*- and R-space spectra for the two solid standards and the four solutions are shown in Fig 3. The small amount of replacement of water molecules by chloride ions upon increasing NiCl_2_ concentration is clearly shown by the phase shift in the imaginary part of the EXAFS Fourier transform (arrow in Fig 3c). In the final analysis, Ni(II) coordination was constrained to be 6, consistent with the result of octahedral configuration obtained from XANES data; the fitting results are listed in Table 2. For all solutions, the refined Ni-O bond distances are 2.07(2) Å, which is consistent with earlier studies [31,37]. For the 1 and 2 mol∙kg^-1^ NiCl_2_ solutions, the fully hydrated ([Ni(H_2_O)_6_]^2+^) model reproduced well the experimental *k*-space and R-space EXAFS spectra. The effect of replacing one of the water molecules with a chloride ligand on the fit quality (reduced chi-square, χ^2^ [38]) of the 2 mol∙kg^-1^ NiCl_2_ solution is illustrated in Fig 4a. The absolute minimum of χ^2^ is located at 0 chloride, and the residuals increase slowly with addition of Cl, resulting in a large uncertainty at the 90% confidence level. In contrast, χ^2^ shows a minimum at 0.44 Cl for the 5.05 mol∙kg^-1^ NiCl_2_ corresponding to the “deepest” depression with an uncertainty of +/– 0.23 Cl (Fig 4b). As expected, the Cl^-^ion resides at a longer distance than O (*R*_Ni-Cl_ 2.35(2) Å vs. *R*_Ni-O_ 2.07(2) Å) due to the larger hard-sphere radius. These results are close to those obtained using XRD by Waizumi et al. [25]; for a 5.06 mol∙kg^-1^ NiCl_2_ solution, these authors reported *N*_Ni-O_ = 5.6 ± 0.2 and *R*_Ni-O_ = 2.06(2) Å; and *N*_Ni-Cl_ = 0.4 ± 0.2 and *R*_Ni-Cl_ = 2.37(2) Å.

**Table 2:** Results of Ni EXAFS analysis of the Ni(II) first shell structure under ambient conditions.

		Ni-O interaction			Ni-Cl interaction						
Composition of system	△E_0_(eV)	*N*_O_	*R*_Ni-O_(Å)	$\sigma_{\text{Cl}}^{\text{2}}$× 10^3^	*N*_Cl_	*R*_Ni-Cl_(Å)	$\sigma_{\text{Cl}}^{\text{2}}$× 10^3^	k-range	R-range	R-factor	Ref.
1.00	–3.86(0.55)	6.0^a^	2.07(06)	5.4(6)	-	-	-	2–10	1–5	0.014	This work
2.00	–4.15(0.65)	6.0^a^	2.07(09)	7.1(8)	-	-	-	2–10	1–5	0.021	This work
4.00	–4.90(0.98)	5.8^b^(3)	2.06(1)	4.8(7)	0.2^b^(3)	2.38(1)	8.4(27)	2–10	1–5	0.014	This work
5.05	–3.46(0.93)	5.6^b^(3)	2.08(1)	2.4(5)	0.4^b^(3)	2.35(2)	7.3(21)	2–10	1–5	0.023	This work
NiSO_4_∙6H_2_O^c^	2.68(1.00)	2.0^a^	2.02(3)	3.0^a^				2–10	1–5	0.030	This work
		2.0^a^	2.03(3)	3.0^a^				2–10	1–5	0.030	This work
		2.0^a^	2.13(5)	3.0^a^				2–10	1–5	0.030	This work
NiCl_2_∙6H_2_O^c^	–1.19(1.53)	4.0^a^	2.07(2)	9(2)	2.0^a^	2.36(4)	13.4(42)	2–10	1–5	0.009	This work
3.12^d^	-	5.92	2.07(1)	-	0.08	2.44(1)	-	-	-	-	[21]
5.06^d^	-	5.6(2)	2.06(2)	-	0.4(2)	2.37(2)	-	-	-	-	[25]

The results for 3.12 and 5.06 mol∙kg^-1^ NiCl_2_ solutions using X-ray diffraction [21,25] are also listed. The concentrations are expressed in molality (mol∙kg^-1^). Uncertainty limits are given in parentheses.

^a^ Value fixed (not optimized) during refinements.

^b^ Sum of number of O and Cl fixed to be equal to 6.

^c^ Solid standard.

^d^ Concentration in molarity (mol∙L^-1^) in references [21,25], converted to molality (mol∙kg^-1^).

### UV-Vis spectroscopy investigation

#### Qualitative analysis of UV-Vis spectra

**Fig 5:**
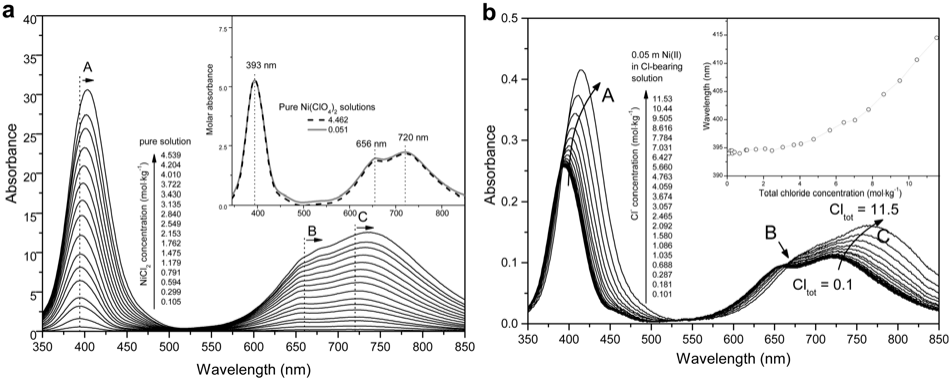
Background-subtracted UV-Vis spectra of (a) NiCl_2_-H_2_O system with salt concentration range from 0.1 to 4.5 mol∙kg^-1^ at room temperature; background subtracted molar absorbance of Ni(ClO_4_)_2_ solutions with 0.05 and 4.46 mol∙kg^-1^ salt concentration are shown in inset; and (b) NiCl_2_-MgCl_2_-H_2_O system, for a constant NiCl_2_ concentration of 0.05 m and MgCl_2_ concentrations from 0 to 5.7 m. The inset show the location of the band at ~400 nm as a function of total Cl concentration.

The baseline-corrected room-temperature spectra (Raw spectral data was collected in S9 and S10 Tables) for both sets of solutions, one with systematically increasing NiCl_2_ concentration and the other containing constant Ni(II) (0.05 mol∙kg^‒1^) but systematically increasing the MgCl_2_ concentration, are presented in Fig 5a and 5b, respectively. The spectra show two characteristic absorption features at 350–500 and 600–800 nm. With increasing NiCl_2_ concentration, the absorbance increases and the spectra display a systematic red-shift: the band in the range of 350–500 nm shifts from ~393 to ~405 nm (arrow A in Fig 5a), and the bands in the 600–800 nm range (arrows B and C in Fig 5a) shifts from ~720 to ~737 nm and ~656 to ~670 nm, respectively. These features become more pronounced for the Ni-Mg-Cl system at higher MgCl_2_ concentration, with the ~393 nm band shifting up to ~415 nm (inset in Fig 5b) and the ~720 band shifting to ~770 nm. The shoulder at ~654 nm becomes weaker (arrow B in Fig 5b, almost vanishing) at the higher chloride concentration. To check the effect of ionic strength on the UV-Vis spectra, solutions of 0.05 and 4.46 mol∙kg^-1^ Ni(ClO_4_)_2_ were measured (inset in Fig 5a). No shift of the peak position was observed, and the molar absorbance coefficients remained nearly constant. Deviations from the Beer-Lambert law at high salinity [75,40] can be due to the influence of the dielectric constant of the medium on the absorption of light by a particular complex [41], or to changes in the electronic structure of the complex induced by changes in the structure of the solvent and in the outer coordination shells of the complex. These effects can be difficult to quantify. The Ni(II) perchlorate data in Fig 5a, however, clearly indicate that the structure of the Ni(II) aqua ion is not sensitive to changes in solvent structure as the salt concentration changes from 0.05 to 4.5 mol∙kg^-1^; this stable structure was also confirmed by the neutron diffraction study of Newsome et al. [42]. Consequently, the Beer-Lambert law appears to be obeyed up to high salinity in this system.

Ligand field theory can be used to describe the electronic absorption spectra of first row transition metal complexes. For the [Ni(H_2_O)_6_]^2+^ species at room temperature, the bands at 800–550 nm and ~500–350 nm are assigned to the ^2^A_2g_→^3^T_1g_ and ^3^A_2g_→^3^T_1g_(P) transitions of the octahedral structure, respectively [10]. The spin-orbit coupling that mixes the ^3^T_1g_(F) and ^1^E_g_ states results in the ^3^A_2g_ to ^3^T_1g_ transition band in the 800–550 nm range being split with a main peak at ~720 nm and a shoulder at ~656 nm [43], which is consistent with a splitting of approximately 18 eV (~68 nm) observed in the Ni L_2,3_-edge XANES spectra [17].

The peak shift observed as a function of NiCl_2_ and MgCl_2_ concentrations reflects the progressive replacement of a H_2_O ligand by a chloride ion around Ni(II) within the first coordination shell, because the *d*-*d* splitting caused by the chloride ion is at lower energy than that produced by water [10]. Since the spectral changes observed via UV-Vis are mainly related to inner-sphere complexing, we performed a quantitative treatment of the UV-Vis dataset to retrieve species concentrations and thermodynamic properties of the Ni(II)-chloride complexes.

#### Speciation model and activity coefficients calculations for quantitative analysis

The quantitative treatment was based on the Beer-Lambert law, following the approach developed by Brugger et al. [13] and implemented in the BeerOz program [39]. The analysis deconvolutes the measured matrix of absorbances into spectra for individual absorbing species (molar absorptivity coefficients) present in solutions, and a matrix of concentrations of these species in each solution. The species concentrations are constrained by a thermodynamic model (mass action and mass balance equations) so that the analysis refines formation constants (log_10_ *K*) of aqueous Ni(II)-chloride complexes rather than actual species concentrations. The spectral dataset was restricted to the wavelengths over which peaks are present (350–550 nm and 580–850 nm). Prior to the analysis of the spectra, the usual molarity scale-based Beer’s law was converted to a molality absorbance by dividing a transfer factor, *f*, defined as a function of density of electrolyte [13,39], which was calculated in present study by the method as described in Zhang et al. [14].

**Fig 6:**
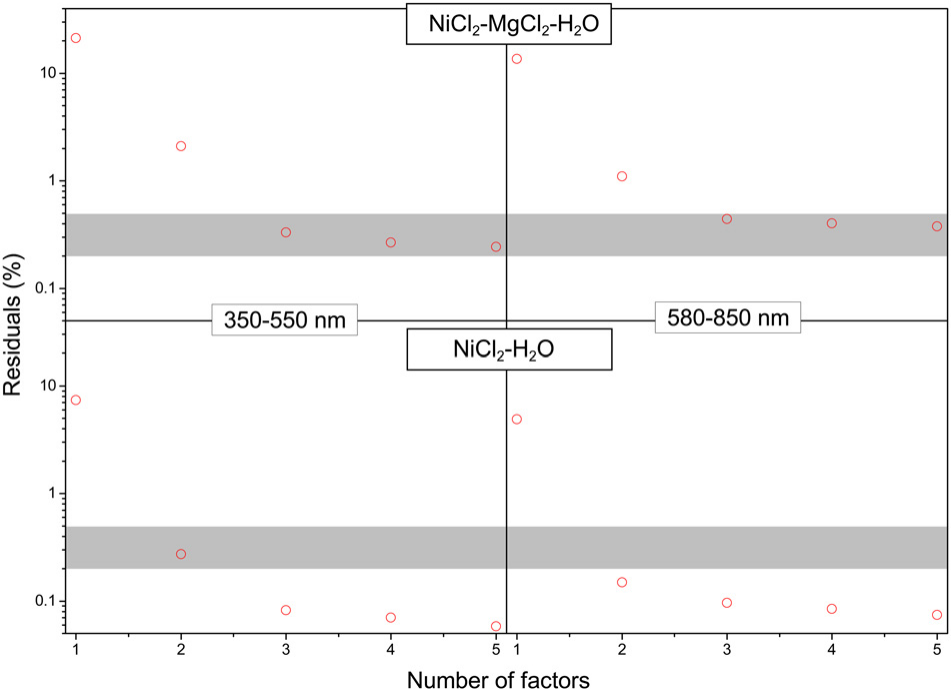
Principal component analysis results for NiCl_2_-H_2_O and Ni-MgCl_2_-H_2_O system in 350–550 nm and 580–850 nm absorbance bands, respectively. The circle is calculated by 100 ×$\sqrt{\frac{{\left(x^{\text{calc}}-x^{\text{meas}}\right)}^{2}}{x^{\text{meas}}}}$, where *x* ^calc^ and *x* ^meas^ are the model and observed value, respectively. The gray area stands for the level of experimental error of 0.2%-0.5%.

Based on the analysis of XAS data above, two main Ni(II) species are present in NiCl_2_ solutions: the fully hydrated aqua ion and the monochloro complex. Principal component analysis (PCA) [44] confirms that two species are required to explain the UV-Vis data for the pure NiCl_2_ solutions within experimental error (Fig 6). In contrast, three species are required to explain the NiCl_2_-MgCl_2_-H_2_O system dataset (Fig 6), suggesting that [NiCl_2_]^0^ may be present in the solutions with the highest ionic strength. This is also consistent with the XAS results of Tian et al. [5]; consequently, the analysis was conducted using the Ni^2+^, NiCl^+^ and [NiCl_2_]^0^ species. The minor species H^+^ and [HCl]^0^ were also included in the speciation model, with the formation constant of the neutral ion pair, [HCl]^0^, taken from the result of Sverjensky et al. [45] (Table 3).

**Table 3:** The solubility product of solid phase in MSE and Pitzer model and the formation constant of NiCl^+^ and [NiCl_2_]^0^ species determined in this study.

		log_10_ *K*/log_10_ *K*_sp_			
Phase	Reaction	MSE	Pitzer	HSC§	Ref.
**Solid**	MgCl_2_∙6H_2_O = Mg^2+^ + 2Cl^-^+ 6H_2_O	4.4 (+0.2/–0.3)	4.5 (+0.1/–0.2)	-	This work
		-	4.307* £	-	[46]
		-	-	4.455	[47]
	NiCl_2_∙6H_2_O = Ni^2+^ + 2Cl^-^+ 6H_2_O	3.2 (+0.1/–0.2)	3.1 (+0.2/–0.1)	-	This work
		3.133	-	3.180	[48]
	NiCl_2_∙4H_2_O = Ni^2+^ + 2Cl^-^+ 4H_2_O	3.9 (+0.1/–0.1)	3.2 (+0.3/–0.1)	-	This work
		3.985	-	3.713	[48]
**Phase**	**Reaction**	**MSE**	**Others**	**HKF**	**Ref.**
**Aqueous**	H^+^ + Cl^-^ = HCl(aq)			–0.711	[45]
	Ni^2+^ + Cl^-^ = NiCl^+^	0.09 (–0.05/+0.03)		-	This work
				–0.43	[10]
			–0.5		[49]
			–1.31 (+0.07/–0.07)		[12]
			–2.0 (+0.2/–0.2)		[7]
	Ni^2+^ + 2Cl^-^ = [NiCl_2_]^0^	–6.6 (–0.5/+0.4)			This work
				–0.95	[10]
			–4.1 (+0.4/–0.4)		[12]
			–4.5 (+0.5/–0.5)		[7]

The association constant of [HCl]^0^ aqueous species used in the calculation is also listed.

Uncertainty limits are given in parentheses.

* The solubility products were converted from mole fraction-scale in the literatures to molality-scale.

^£^ Value was calculated by Pitzer−Simonson−Clegg (PSC) model [46].

^§^ Values were calculated by HSC software (H = enthalpy, S = entropy, C = heat capacity) in literature [48].

^¶^ The activity coefficient model assumed *γ*_ion_ = *γ*_*±*_,_ion_.

In order to calculate the distribution of species in concentrated NiCl_2_ solutions, activity coefficients need to be available for all the species in the model. The “b-dot” equation developed by Helgeson and Kirkham [50] is a popular method for calculating activity coefficients, used for example by Liu et al. [10] in their UV-Vis study of Ni(II)-chloride complexes in chloride brines up to high temperature. This model is well suited for relatively dilute solutions (ionic strength ≤ 2 molal), though some studies extended the approach to higher salinities (10,1–15,40). The ion-interaction Pitzer model [51] is a popular semi-empirical approach used to describe the thermodynamics of concentrated electrolyte solutions. Originally, Pitzer’s model assumed full dissociation of electrolytes, but several authors [52–54] have introduced speciation-based extensions to the theory. In the present study, we implemented the speciation-based mixed-solvent electrolyte (MSE) model [55–57]. This comprehensive and self-consistent model has the following advantages: (i) it is a speciation-based model, taking complexation reactions explicitly into account; (ii) it provides a bridge to the Helgeson-Kirkham-Flowers (HKF) approach [50,58], so that predictions can be expanded over a wide range of pressures and temperatures; (iii) it covers the full concentration range from highly dilute to the solubility limit. The standard-state properties calculated from the model of Helgeson and Kirkham [59] are based on the molality concentration scale and on the infinite-dilution reference state and result in large excess properties in concentrated solutions, whereas the activity coefficients in the MSE framework are based on the mole fraction scale and are symmetrically normalized (i.e., unit activity coefficients for x_*i*_ = 0 and x_*i*_ = 1).

In the MSE model, the activity coefficient of any aqueous species *k* is expressed as a sum of three terms,

$$\text{ln}\gamma_{k}=\text{ln}\gamma_{k}^{\text{LR}}+\text{ln}\gamma_{k}^{\text{MR}}+\text{ln}\gamma_{k}^{\text{SR}}$$ (1)

where LR represents the long-range electrostatic interactions between ions at low concentrations, calculated using the Pitzer-Debye-Hückel expression [55,60]; SR is the short-range contribution resulting from intermolecular interactions, calculated by the UNIQUAC equation [61]. As ions are the dominant species in this study, the short-range term can be neglected [62]. MR is an additional symmetrical second virial coefficient-type (middle-range) term, which represents primarily ionic interactions (i.e., ion-ion and ion-molecule) that are not accounted for by the LR term. The MR term is given by [55]

$$\text{ln}\gamma_{k}^{\text{MR}}=\sum_{i}\sum_{j}x_{i}x_{j}B_{ij}(I_{x})-\left(\sum_{i}n_{i}\right)\sum_{i}\sum_{j}x_{i}x_{j}\frac{\partial B_{ij}(I_{x})}{\partial n_{k}}-2\sum_{i}x_{i}B_{ik}(I_{x})$$ (2)

where *i* and *j* represent aqueous species (ions or molecules), *x* is the mole fraction, *n* is the mole number and *B*_*ij*_(*I*_*x*_) are binary interaction parameters, which are assumed to be symmetric, i.e., *B*_*ij*_(*I*_*x*_) = *B*_*ji*_(*I*_*x*_), and *B*_*ii*_(*I*_*x*_) = *B*_*jj*_(*I*_*x*_) = 0, and dependent on the ionic strength, with

$$B_{ij}(I_{x})=b_{ij}+c_{ij}\exp(-\sqrt{I_{x}+a_{1}})$$ (3)

where

$$b_{ij}=b_{0,ij}+b_{1,ij}T+\frac{b_{2,ij}}{T}+b_{3,ij}T^{2}+b_{4,ij}\text{ln}T$$ (4)

$$c_{ij}=c_{0,ij}+c_{1,ij}T+\frac{c_{2,ij}}{T}+c_{3,ij}T^{2}+c_{4,ij}\text{ln}T$$ (5)

For the final fit, the MSE MR parameters *b*_0,*ij*_ and *c*_0,*ij*_ (Table 4), the formation constants log_10_ *K*(NiCl^+^) and log_10_ *K*([NiCl_2_]^0^), and the molar absorptivity coefficients for NiCl^+^ and [NiCl_2_]^0^ were optimized by using non-linear least squares [39] to minimize the residual function *χ* ^2^,

$$\begin{array}{l} \chi^{2}=w_{1}\chi_{\text{UV}-\text{Vis}}^{2}+w_{2}\chi_{\text{Mean Activity}}^{2}+w_{3}\chi_{\text{Solubility}}^{2}\\ =w_{1}\sum_{v=1}^{V}\left[\sum_{u=1}^{U}{\left({\text{A}}_{vu}^{\text{exp}}-{\text{A}}_{vu}^{\text{calc}}\right)}^{2}\right]+w_{2}\sum_{l=1}^{L}{\left(\gamma_{\pm ,l}^{\text{exp}}-\gamma_{\pm ,l}^{\text{calc}}\right)}^{2}+w_{3}\sum_{i=1}^{I}{\left(K_{\text{sp,i}}^{\text{exp}}-K_{\text{sp},i}^{\text{calc}}\right)}^{2} \end{array}$$ (6)

**Table 4:** MSE middle-range ion interaction parameters in this study.

Species *i*	Species *j*	*b*_0,*ij*_	*c*_0,*ij*_
Ni^2+^	Cl^-^	–54.9768	69.0819
NiCl^+^	Cl^-^	–28.5398	-
Ni^2+^	Mg^2+^	–33.8995	50.9434
NiCl^+^	Mg^2+^	–6.8626	-
[NiCl_2_]^0^	Mg^2+^	–0.0984	-
Mg^2+^	Cl^-^	–82.4238	112.1769

The experimental data consisted of the UV-Vis spectra data for 16 solutions with NiCl_2_ concentrations ranging from 0.1 to 4.53 mol∙kg^-1^, and for 23 solutions with a constant Ni(II) concentration (~0.05 mol∙kg^-1^) with MgCl_2_ concentrations ranging from 0 to 5.72 mol∙kg^-1^ ($\chi_{\text{UV}-\text{Vis}}^{2}$), together with the experimental mean activity coefficient data of NiCl_2_ solutions at room temperature ($\chi_{\text{Mean Activity}}^{2}$) [63], and the solubility data in the NiCl_2_-MgCl_2_-H_2_O ternary system ($\chi_{\text{Solubility}}^{2}$) [64]. In Eq (6), *V* is the total number of wavelengths at which measurements were made, and *U* is the number of solutions. ${\text{A}}_{vu}^{\text{exp}}$and ${\text{A}}_{vu}^{\text{calc}}$ are the measured and calculated absorbance at wavelength *v* for solution *u*. *w*_1_, *w*_2_ and *w*_3_ are weighting factors and are set as 0.5, 1.0 and 1.0, respectively, in the present study. $\gamma_{\pm ,l}^{\text{exp}}$and $\gamma_{\pm ,l}^{\text{calc}}$ are the experimental and calculated mean activity coefficient of solution *l*; the experimental activity coefficients show a deviation from ideal behavior at high concentration due to the formation of Ni(II)-chloride complexes [63], and can be described as a combination of an ideal stoichiometric activity coefficient, and the effect of complexation. Aparicio and Elizalde [65] calculated the experimental mean stoichiometric activity coefficient for an electrolyte containing a significant amount of complexes (ZnCl_2_ solutions), and their approach is used here:

$$\gamma_{\pm ,l}^{\text{calc}}=\frac{{\left\{{\left(m_{+}\right)}^{\upsilon +}{\left(m_{-}\right)}^{\upsilon -}\right\}}^{1/\upsilon }{\left\{{\left(\gamma_{+}\right)}^{\upsilon +}{\left(\gamma_{-}\right)}^{\upsilon -}\right\}}^{1/\upsilon }}{m{\left(\upsilon_{+}^{\upsilon +}\upsilon_{-}^{\upsilon -}\right)}^{1/\upsilon }},$$ (7)

where *υ*_+_ and *υ*_−_ are the number of Ni^2+^ and Cl^-^ions, whose activity coefficients are *γ*_+_ and *γ*_−_ calculated by the MSE model, and *m*_+_ and *m*_—_are their respective equilibrium concentrations in molality; *υ* = *υ*_+_+*υ*_−_; and *m* is the conventional total NiCl_2_ concentration (in molal). The Mg^2+^-Cl^-^mixing parameters were refined from the mean stoichiometric activity and water activity data [66] (Table 5). *K*_sp_ in Eq (6) represents the solubility product of the solid phase M_*v*+_X_*v*–_∙*n*H_2_O, which is an equilibrium reaction with aqueous species and given by

$${\text{M}}_{v+}{\text{X}}_{v-}\cdot n{\text{H}}_{\text{2}}\text{O}=v_{+}{\text{M}}^{z+}+v_{-}{\text{X}}^{z-}+n{\text{H}}_{\text{2}}\text{O}$$ (8)

*K*_sp_ is calculated by

$$K_{sp}=a_{{\text{M}}^{z+}}^{v+}a_{{\text{X}}^{z-}}^{v-}a_{{\text{H}}_{\text{2}}\text{O}}^{n}={\left(\gamma_{{\text{M}}^{z+}}m_{{\text{M}}^{z+}}\right)}^{v+}{\left(\gamma_{{\text{X}}^{z-}}m_{{\text{X}}^{z-}}\right)}^{v-}$$ (9)

where *a* is the activity, *γ* is the activity coefficient calculated by Eq (1), and *m* is the concentration in molality. In this work, the solid phases MgCl_2_∙6H_2_O and NiCl_2_∙*n*H_2_O (*n* = 4, 6) in the ternary system NiCl_2_-MgCl_2_-H_2_O at room temperature are involved for reproducing experimental solubility data [64].

**Table 5:** Experimental data used for parameters estimation.

Systems	data type	Ref.
MgCl_2_-H_2_O	$a_{{\text{H}}_{\text{2}}\text{O}}$ *, *γ*_±_	[66]
NiCl_2_-H_2_O	*γ*_±_	[63]
MgCl_2_-NiCl_2_-H_2_O	SLE**	[64]

* Water activity data

** Solid liquid equilibrium data

Due to the low concentration of [NiCl_2_]^0^ present in the solution, correlations among concentration and molar absorptivity coefficients [39,40], and the large number of parameters involved in the regression, a step-wise approach was required to obtain a self-consistent solution for the NiCl_2_-MgCl_2_-H_2_O system: **1)** refine the molar absorbance of the NiCl^+^ species, the formation constant of NiCl^+^, and the mixing parameters for the Ni^2+^-Cl^-^and NiCl^+^-Cl^-^interaction based on the NiCl_2_-H_2_O system spectrophotometric data. **2)** The molar absorbance and formation constant of [NiCl_2_]^0^ species were fixed using the results of step 1). **3)** We then refined the molar absorbance and formation constant for [NiCl_2_]^0^ species, as well as the $b_{0,{\text{NiCl}}_{\text{2}},{\text{Mg}}^{\text{2}+}}$ mixing parameter based on the NiCl_2_-MgCl_2_-H_2_O system spectrophotometric data.

#### Results of the quantitative analysis of UV-Vis data

**Fig 7:**
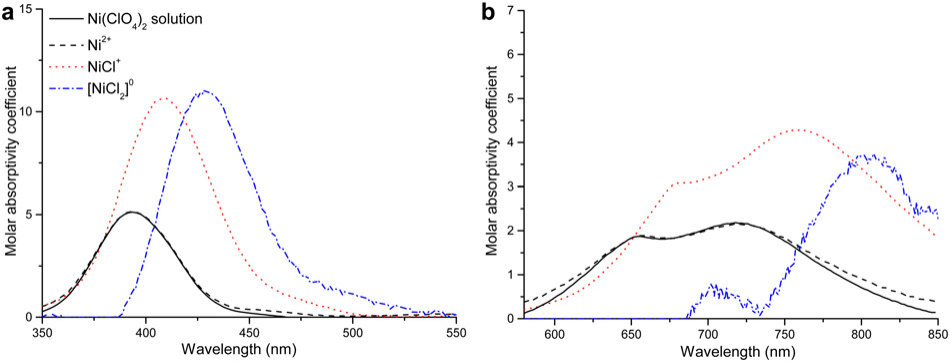
Molar absorptivity spectra of individual Ni(II)-chloride species obtained from the analysis of two absorptive band (left, 350–550 nm and right 580–850 nm) spectroscopic data for NiCl_2_-H_2_O and NiCl_2_-MgCl_2_-H_2_O systems at room temperature. The molar spectrum for Ni(ClO_4_)_2_ solution at room temperature is plotted as solid line for comparison.

The analysis provides a good agreement between experimental and calculated spectra; for the 350–550 nm range, the largest differences (in absorbance unit) were 0.05 (NiCl_2_-H_2_O system) and 0.07 (NiCl_2_-MgCl_2_-H_2_O system); and for the 580–850 nm range, 0.012 and 0.006, respectively. The regressed MSE ion interaction parameters are presented in Table 4, and the optimized formation constants of Ni(II)-chloride complexes and the solubility products of solids obtained from solid-liquid equilibrium data and spectral data are reported in Table 3 together with the comparison of log_10_ *K*_sp_/log_10_ *K* values at 25°C from this work and the literature [7,10,12,46,47,48,], respectively. The uncertainties of formation constants for NiCl^+^ and [NiCl_2_]^0^ at the 90% confidence level are also listed; they are derived from residual maps as described in the literature [39]. The molar absorptivity spectra of individual Ni(II)-chloride complexes are shown in Fig 7. The molar absorptivity spectra of individual Ni(II)-chloride complexes show a shift to lower energy (red-shift) with increasing substitution of chloride ions. We also conducted a fit in which we refined the molar absorptivity coefficients for the Ni(II) species; the refined the molar absorptivity spectrum is close to the spectrum fixed from measurement of the Ni(ClO_4_)_2_ solution, indicating that the analysis method is reliable. There is a large increase in molar absorptivity from Ni^2+^ to NiCl^+^; the change in intensity between NiCl^+^ and [NiCl_2_]^0^ was model-sensitive, which is attributed to the low concentration of this complex in the studied solutions. For the 580–850 nm region (Fig 7), the analysis only reveals a red-shift for the molar absorbance spectrum of [NiCl_2_]^0^, but failed to provide a realistic molar absorptivity spectrum for this complex. The changes of the calculated spectra of each individual Ni(II) species are analogous with the results obtained for minor amounts of Ni(II) in NaCl solutions at elevated temperatures [10].

**Fig 8:**
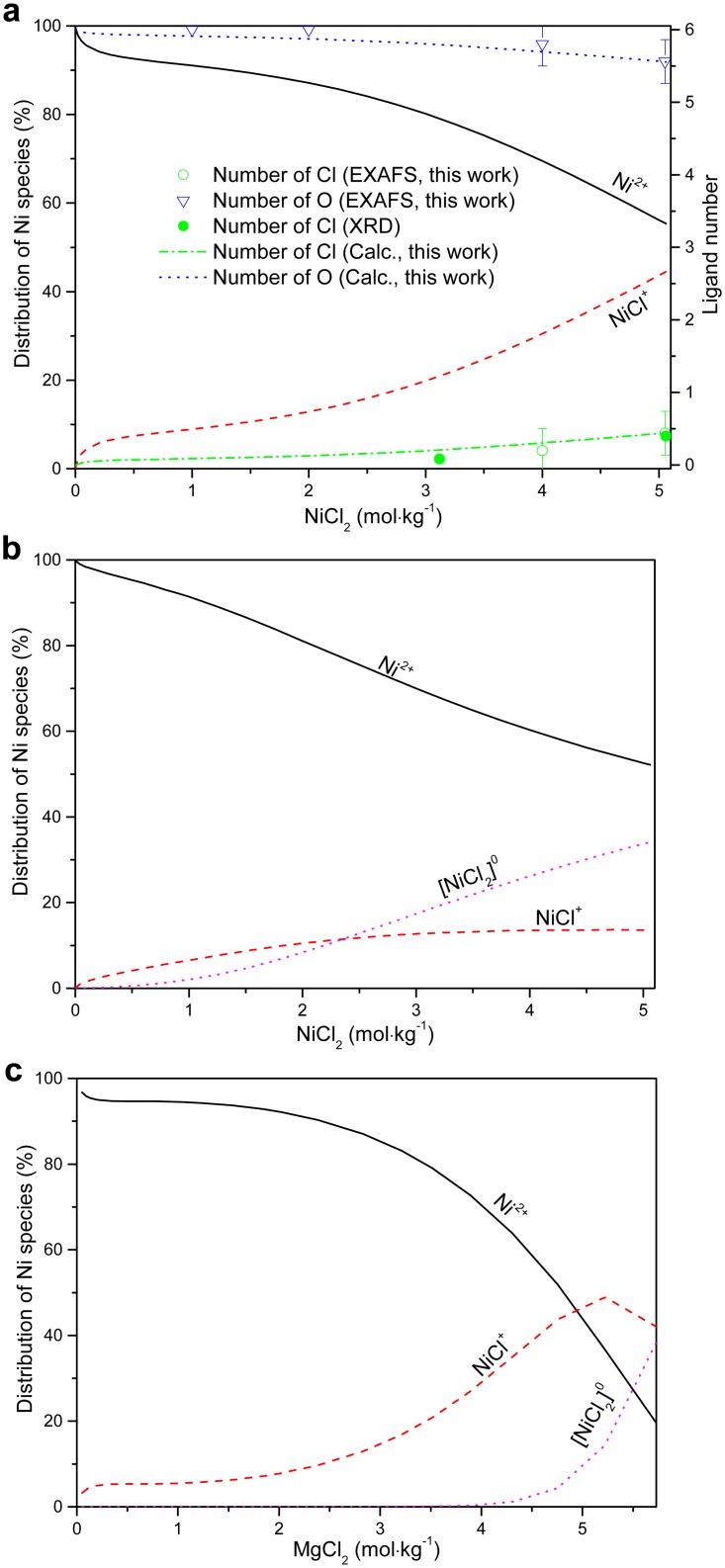
Distribution of Ni(II) species in NiCl_2_-H_2_O (a, b), and NiCl_2_-MgCl_2_-H_2_O (c) solutions and average number of ligand in the first shell of Ni^2+^ as a function of NiCl_2_ solution concentration. In (a) and (c), the distributions of species are calculated with the formation constant and MSE parameters determined in this study (all lines); in (b), the distributions of species was calculated for NiCl_2_ solutions using the model and parameters from literature [10]; In (a), the calculated average numbers of ligands in the first shell of Ni^2+^ are compared with the number of ligands extracted by EXAFS (Cl ligand, empty circles with error bar; O ligand, triangles with error bar) and XRD [21,25] (Cl ligand, filled circles) analysis are shown for comparison; and (b) the result using the model and parameters from literature [10].

**Fig 9:**
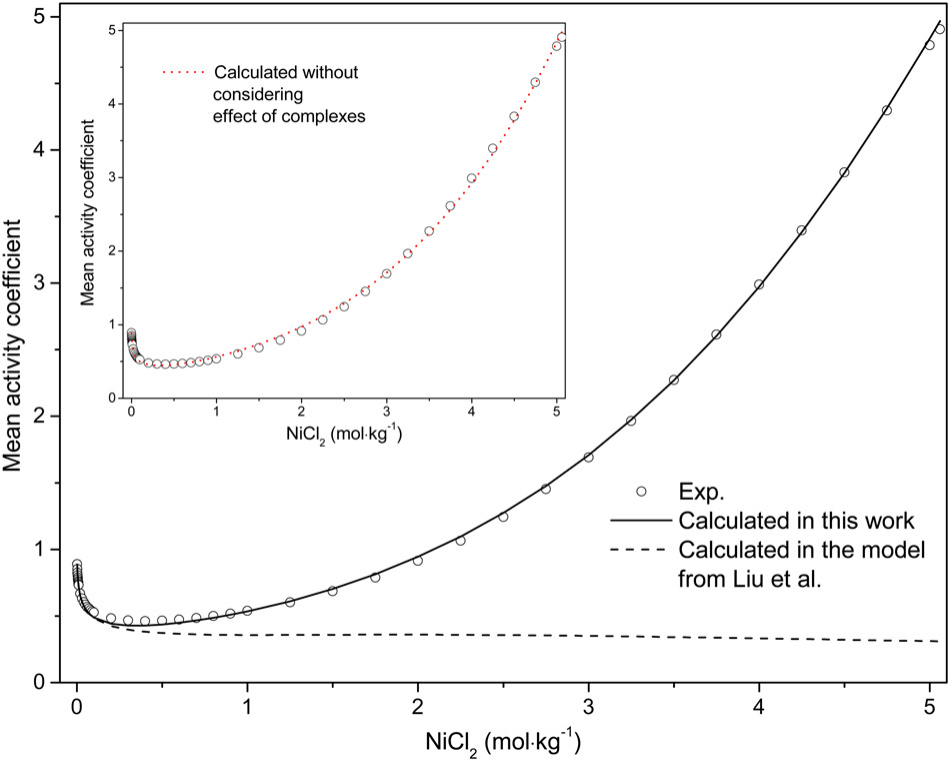
Comparison of experimental and calculated mean activity coefficient for NiCl_2_ solution at room temperature. The line is calculated ones using the method of this work; the dash line represents the calculated results using the method from literature [10]; the circle are literature values [63]. The dot line of inset is the mean stoichiometric activity coefficient, namely, without considering effect of complexes in system, with the parameters derived in present model.

The formation constant of NiCl^+^ in this work is larger than that reported in the potentionmetric study of Libus and Tialowska [49] and in the spectrophotometric studies of Bjerrum [7], Liu et al. [10] and Paatero and Hummelstedt [12], whereas that of the neutral [NiCl_2_]^0^ complex is more negative than that reported by Liu et al. [10] (Table 3) but closer the values from Bjerrum [7] and Paatero and Hummelstedt [12]. The distributions of absorbing species in the NiCl_2_-H_2_O and NiCl_2_-MgCl_2_-H_2_O solutions calculated with the new model show a larger percentage of the NiCl^+^ complex in the NiCl_2_-MgCl_2_-H_2_O solutions (higher Cl/Ni ratio) compared to the NiCl_2_-H_2_O solutions, implying a stronger association in the former solutions (Fig 8a and 8c). In addition, the neutral complex [NiCl_2_]^0^ can account up to ~38% Ni(II) in the MgCl_2_ solutions. For comparison, we calculated the distribution of Ni(II) species in NiCl_2_-H_2_O solutions using Liu et al. [10]’s model, in which the activities of the species are calculated using the “b-dot” equation (Fig 8b). The agreement is good at low NiCl_2_ concentrations (< 1 mol∙kg^-1^ NiCl_2_). However, the predictions diverge with increasing electrolyte concentration, with Liu et al. [10]’s model showing the neutral complex, [NiCl_2_]^0^, playing an important role (up to ~30%) in pure NiCl_2_ solutions at high concentrations, while our model and UV-Vis data suggest that no significant [NiCl_2_]^0^ is present (Fig 8a). This apparent contradiction is the result of the poor description of activity-composition relationships in highly saline solutions in the HKF model. The experimental mean activity coefficient of NiCl_2_ solutions is compared to the values calculated using the MSE parameters and the model of Liu et al. [10] in Fig 9. The mean activity coefficient calculated in this work agrees well with the experimental data [63], while Liu et al. [10]’s model only reproduces the data at low NiCl_2_ concentrations (≤ 0.5 mol∙kg^-1^), with increasingly significant deviations occurring with increasing salt concentrations.

**Fig 10:**
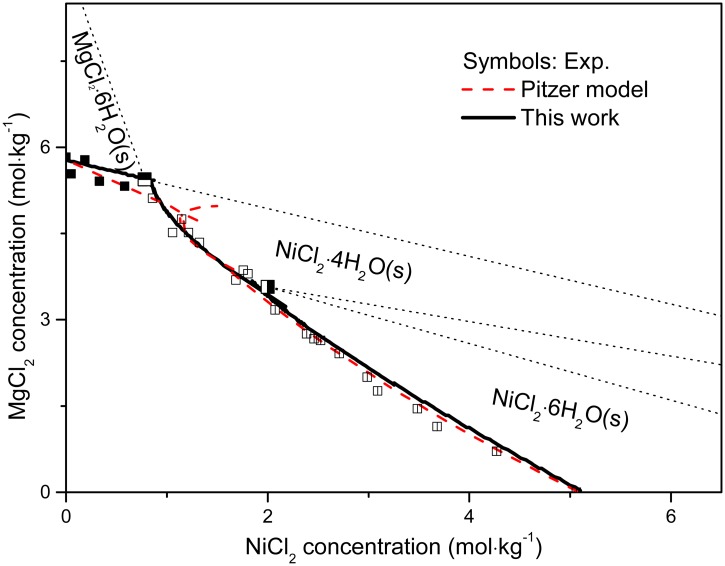
Calculated solubility isotherms (solid line by MSE model in this work and dashed line by Pitzer model) compared with experimental values (symbols: ■, MgCl_2_∙6H_2_O; ◪, MgCl_2_∙6H_2_O + NiCl_2_∙4H_2_O; □, NiCl_2_∙4H_2_O; ◨, NiCl_2_∙4H_2_O + NiCl_2_∙6H_2_O; ◫, NiCl_2_∙6H_2_O, data from literature [64]) at room temperature.

**Fig 11:**
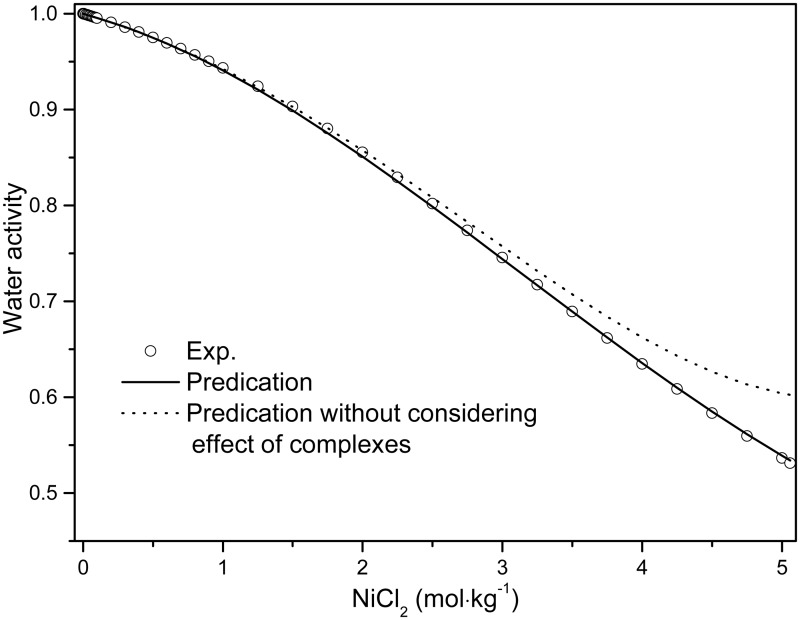
Comparison experimental and predicted water activity for NiCl_2_ solution at room temperature. The line is predicted one in this work and the dot line represents the prediction one without considering effect of complexes in system, corresponding to the dot line in Fig 9; the circles are literature values [63].

The solubility products from this work are mostly in agreement with the literature values [46–48]. The solubility isotherms calculated using the parameters from Tables 3 and 4 are displayed in Fig 10, and are consistent with the experimental values [64]. If ion association is neglected for the NiCl_2_-MgCl_2_-H_2_O ternary system, the results are unsatisfactory as shown in the dashed line of Fig 10 using the Pitzer completely dissociated model (see [47,67] for details of calculation procedure), although the solubility products of the solid phases are comparable in both models as tabulated in Table 3. Furthermore, the predicted water activity in the NiCl_2_-H_2_O system in this work is in excellent agreement with the literature value [63] (Fig 11). In the absence of Ni(II)-chloride complexing, the calculated water activity for pure NiCl_2_ solutions deviated from the experimental values at high salt concentration (dotted line in Fig 11). The deviation occurs at higher concentration for NiCl_2_ solutions than that for ZnCl_2_ solutions (~2.5 mol∙kg^-1^ vs ~0.05 mol∙kg^-1^, respectively [65]); this is consistent with the smaller extent of chloro complex formation in Ni(II) chloride solution relative to Zn(II) chloride solutions [68–70]. We also attempted to fit the experimental mean activity coefficient of NiCl_2_ solutions without considering the NiCl^+^ complex (dotted line in inset of Fig 9), with no significant decrease in fit quality.

The results of our speciation calculations confirm that the fully hydrated Ni(II) species is dominant in NiCl_2_-H_2_O solutions, with only a small amount of chloride anions replacing water within the first octahedral shell even in saturated NiCl_2_ solutions under ambient conditions. This indicates that the capacity of Ni(II) to bind chloride is much weaker that Zn(II) [16,70], Cu(II) [13–15] and Co(II) [4]. The average numbers of inner-sphere chloride and oxygen (water molecule) ligands calculated based on our thermodynamic speciation model agree with the experimental results from EXAFS and XRD experiments [21,25] (Fig 8a). Note that the low proportion of NiCl^+^ (~30%, i.e. 0.3 Cl on average around Ni atoms) present in solution was detected by UV-Vis spectroscopy (red-shift) and XANES (subtle decrease of the intensity of the white line), but can easily be overlooked in the EXAFS data. Therefore, accurate results require the integration of results from a range of techniques and careful error analysis. We note that the apparent discrepancies in the literature—with some studies claiming no inner-sphere complexing in concentrated NiCl_2_ solutions—are related to poor consideration of errors. In addition, the molecular dynamics simulations of Xia et al. [26], used to dismiss the formation of ion pairs in concentrated NiCl_2_ solutions, are inconclusive because the simulation times (19 ps) were short relative to the ligand exchange rates (*k*_ex_), e.g. 3 × 10^4^ to 10^2^ s^-1^ for M-Cl exchange (M = Au(III), Zn(II), Cd(II) [71–72]; 3.2 × 10^4^ s^-1^ for Ni-H_2_O exchange at room temperature ([73]). Aziz et al. [17] observed a geometrical distortion of the Ni(II) coordination sphere with increasing NiCl_2_ concentration (0.05 to 1.5 mol∙L^-1^) on the basis of L_2,3_-edge XANES spectra. They interpreted this distortion to reflect solvent-shared ion pairs rather than inner-sphere complexes, on the basis of a comparison with an unspecified NiCl_2_∙*n*H_2_O_(s)_ solid in vacuum. However, in view of the uncertainty on the hydration state of the standard, and the low concentrations of the inner-sphere complex even in concentrated NiCl_2_ solutions, Aziz et al. [17]’s L_2,3_-edge XANES data are consistent with the speciation proposed in this study. Indeed, more recent L-edge XANES spectroscopy studies have emphasized the sensitivity of this method to small amounts of ion pairing (e.g., [74]).

## Conclusions

The Ni(II)-chloride interaction in aqueous solutions of NiCl_2_-H_2_O (Cl:Ni = 2) and NiCl_2_-MgCl_2_-H_2_O systems (Cl:Ni from 2 to 230) was systematically investigated using XAS and UV-Vis spectroscopy at room temperature. All the evidence indicates that there is no large structural change (e.g., from octahedral to tetrahedral configuration) in these systems up to the solubility limit. The analysis confirms that the hexa-aqua Ni(II) complex is the major complex in all studied solutions. However, the subtle changes of XAS and UV-Vis spectra as a function of salinity indicate that a small amount of Ni(II)-chloride complexation occurs, with up to 0.44(23) Cl in the first coordinated shell of Ni(II) at 5.05 mol∙kg^-1^ at a Ni-Cl distance of 2.35(2) Å. At high Cl:Ni ratio, small amounts of the [NiCl_2_]^0^ complex are also present. These results are consistent with existing literature.

We developed a self-consistent thermodynamic model, based on the comprehensive MSE framework, for predicting the speciation, mean activity coefficients, water activities, and mineral solubilities in Ni-Cl-Mg-H_2_O solutions up to the solubility limits. The stability of the Ni(II)-chloride complexes was retrieved mainly from a quantitative analysis of the UV-Vis spectroscopic data. The obtained speciation model is consistent with existing XAS and XRD data, and can easily be extended to include further components.

## Supporting Information

S1 Table Compositions (in molal) of the 18 NiCl_2_-H_2_O solutions measured by UV-Vis. (TXT)

S2 Table Compositions (in molal) of the 23 NiCl_2_-MgCl_2_-H_2_O solutions measured by UV-Vis. (TXT)

S3 Table XAS data for the NiCl_2_·6H_2_O_(s)_ standard. (TXT)

S4 Table XAS data for the NiSO_4_·6H_2_O_(s)_ standard. (TXT)

S5 Table XAS data for the 1 mol·kg^-1^ NiCl_2_ solution. (TXT)

S6 Table XAS data for the 2 mol·kg^-1^ NiCl_2_ solution. (TXT)

S7 Table XAS data for the 4 mol·kg^-1^ NiCl_2_ solution. (TXT)

S8 Table XAS data for the 5 mol·kg^-1^ NiCl_2_ solution. (TXT)

S9 Table Raw absorbance data for the NiCl_2_-H_2_O solutions. (XLS)

S10 Table Raw absorbance data for the NiCl_2_-MgCl_2_-H_2_O solutions. (XLS)
